# Individual and Combined Effects of Legacy and Emerging Contaminants on the Blue Crab *Callinectes sapidus*: Mercury and Bisphenol S as a Case Study

**DOI:** 10.3390/jox16030096

**Published:** 2026-05-29

**Authors:** Jacopo Fabrello, Giovanni Martino Rigodanza, Francesco Boldrin, Federico Caicci, Marco Munari, Valerio Matozzo

**Affiliations:** 1 Department of Comparative Biomedicine and Food Science, University of Padova, Viale Dell’Università’, 16, 35020 Legnaro, Italy; 2 Department of Biology, University of Padova, Via Ugo Bassi 58/B, 35131 Padova, Italy; giovannimartino.rigodanza@studenti.unipd.it (G.M.R.); francesco.boldrin@unipd.it (F.B.); federico.caicci@unipd.it (F.C.); 3 Department of Integrative Marine Ecology, Stazione Zoologica Anton Dohrn SZN, Villa Comunale, 80121 Napoli, Italy; marco.munari@szn.it

**Keywords:** mercury, bisphenols, mixture toxicity, crabs, biomarkers

## Abstract

This study investigated the individual and combined effects of mercury (as HgCl_2_) and Bisphenol S (BPS) on the blue crab *Callinectes sapidus*, focusing on biomarkers measured in hemolymph, gills, and hepatopancreas and ultrastructure (transmission electron microscopy, TEM) observations. Adult males were exposed for seven days to environmentally relevant concentrations of each contaminant, alone and in mixture. Results revealed clear tissue-specific responses. In hemolymph, both contaminants reduced total hemocyte count and affected immune-related parameters, including hemocyte proliferation and enzymatic activities. In gills, mercury significantly decreased total antioxidant capacity, while both contaminants increased lipid peroxidation, indicating oxidative stress. BPS and the mixture stimulated catalase activity, whereas electron transport system activity increased only under combined exposure. In contrast, the hepatopancreas showed limited biochemical alterations, suggesting a higher resilience to medium-term exposure. TEM observations revealed a general decrease in granularity of hemocytes from crabs exposed to BPS and mixture, suggesting cell degranulation. In addition, infiltration of hemocytes into gills was observed in crabs exposed to experimental conditions, suggesting migration of hemocytes from hemolymph to peripheral tissues, while alterations of the microvilli arrangement were detected in digestive cells of BPS-treated crabs. Overall, the findings suggested that hemolymph and gills were more sensitive to contaminant exposure, while the hepatopancreas appears more resistant, at least under the experimental conditions tested. The study also suggests potential non-additive interactions between mercury and BPS.

## 1. Introduction

Mercury (Hg) and bisphenol S (BPS) are two contaminants of environmental concern that have gained significant attention in recent years due to their widespread presence in aquatic environments, including marine ecosystems. Hg has been extensively used in various industrial processes for centuries, despite its well-documented toxicity and environmental persistence [1,2,3,4]. Its unique physicochemical properties have made it valuable in sectors such as chlor-alkali production, electrical equipment manufacturing, and artisanal gold mining [5]. However, the widespread industrial application of mercury has led to significant environmental concerns, particularly in marine-coastal ecosystems, which often serve as the ultimate sink for this contaminant [6]. Hg is considered one of the priority pollutants of global concern due to its persistence, long-range transport, bioaccumulation potential, and conversion into methylmercury, a highly toxic and biomagnifying form readily transferred through aquatic food webs [6]. The chlor-alkali industry, historically one of the largest consumers of mercury, has been a major contributor to environmental mercury loads [7]. Additionally, coal-fired power plants, waste incineration facilities, and metal smelting operations release substantial amounts of mercury into the atmosphere, which eventually finds its way into aquatic systems [8]. The pathways of mercury contamination in marine-coastal environments are multifaceted. Atmospheric deposition is a primary route, with both wet and dry deposition contributing to the mercury burden in coastal waters and sediments [9]. River discharge represents another significant pathway, transporting mercury from inland sources to estuarine and coastal areas [10]. Once in the marine-coastal environment, mercury undergoes complex biogeochemical transformations. Of particular concern is the conversion of inorganic mercury to methylmercury, a highly toxic and bioaccumulative form, by anaerobic microorganisms in sediments [11]. Methylmercury can then enter the food web, biomagnifying through trophic levels and potentially impacting both wildlife and human health [12].

The concentrations of total mercury (THg) in marine ecosystems vary widely depending on the location and the environmental matrices analyzed. In seawater, mercury concentrations range from 0.5 ng/L in the North Sea up to 27,060 ng/L in the Mediterranean Sea (Tunisia) [13]. Sediment concentrations also show great variability, reflecting both natural background and anthropogenic contamination. For example, a minimum level of about 0.0005 mg/kg was found in East China Sea, whereas concentrations increased markedly up to 5330 mg/kg in sediments from the Mediterranean Sea (Italy) [13]. Asnicar et al. [14] recorded Hg concentrations ranging from 20 to 930 μg/Kg in five surface sediment samples collected along a navigation canal in the Lagoon of Venice. In Yuhuan coastal area (China), mean values of Hg concentrations in the surface sediments and surface seawater were 0.05 mg/g and 0.03 mg/L, respectively [15]. A study on the spatial and seasonal distribution of heavy metals in water and sediment samples from three major mangrove sites along the Arabian Sea coast of Pakistan revealed concentrations of Hg in the range of several tens of μg/L [16].

On the other hand, Bisphenol S (BPS) has emerged as one of the common substitutes for Bisphenol A (BPA) in various industrial applications, following concerns about the endocrine-disrupting properties of BPA [17]. BPS is widely used in the production of epoxy glues, thermal paper, sulfonated poly (ether ketone ether sulfone), and as additives in dyes and tanning agents [18]. However, the increasing use of BPS has raised new environmental concerns, particularly regarding its presence and potential impacts in marine-coastal ecosystems [19]. The industrial applications of BPS are diverse and expanding. It is commonly used in the manufacturing of food packaging materials, including can linings and plastic containers [20]. BPS is also utilized in the production of thermal receipt paper, as a flame retardant in electronics, and in various personal care products [21]. These widespread applications have led to significant environmental releases and subsequent contamination of aquatic environments. The pathways of BPS contamination in marine-coastal environments are multifaceted. Wastewater treatment plants (WWTPs) represent a primary source of BPS [22,23], with consequent potential introduction into coastal waters and estuaries. Surface runoff from urban areas also contributes to BPS loading in aquatic environments [22]. Another significant pathway is the degradation of BPS-containing plastic debris in marine environments [24]. In aquatic ecosystems, BPS concentrations ranged from 0.15 ng/L to 12 ng/L, with an average of 2.2 ng/L, in seawater samples from the East China Sea, whereas in sediment samples BPS concentrations ranged from 0.12 ng/g d.w. to 5.4 ng/g d.w., with an average of 0.69 ng/g d.w. [25]. Sun et al. [26] have recently demonstrated that the concentrations of BPS can vary markedly, with values ranging from 156.8 to 1099 ng/L during the wet season and from 85.89 to 859.1 ng/L in the dry season in seawater samples from the Pearl River Estuary of the northern South China Sea. In another study, it was shown that BPS concentrations ranged from 19.9 to 65,600 ng/L in river water and from 0.06 to 45.4 ng/g in sediment samples from Guangzhou, South China [27].

These data suggest a significant presence of both contaminants in aquatic ecosystems, particularly in coastal and estuarine areas. However, the combined effects of legacy and emerging contaminants, such as mercury and BPS, on marine organisms remain largely unexplored. Therefore, this study aims to evaluate the toxic effects of the two contaminants, both individually and in combination, on the blue crab *Callinectes sapidus*. *C. sapidus*, is a crustacean species native to the western Atlantic Ocean [28]. Due to its great adaptability to changing environmental conditions [29,30], *C. sapidus* has become an invasive species in several regions of the world, including the Mediterranean Sea [31]. Once introduced, the blue crab frequently outcompetes native crustaceans, leading to significant disruptions in local biodiversity and also to economic damage [31]. Indeed, its strong predatory behavior, competition for resources, and ability to alter habitats have raised considerable ecological concerns [32].

Generally, the adaptability of an aquatic species to new environments may also depend on its ability to respond to environmental pollution. To investigate crabs’ responses to environmental contaminants, we exposed blue crab specimens for 7 days to Hg and BPS—singly and in combination—and a battery of biomarkers was measured, together with transmission electron microscopy (TEM) observations, in hemolymph, gills and hepatopancreas to preliminarily delineate the species’ degree of resistance to chemical insult. To the best of our knowledge, this is among the first studies investigating the combined effects of mercury and BPS in a marine decapod crustacean species using an integrated approach combining hemolymph biomarkers, oxidative stress parameters, and TEM observations. The study therefore contributes new insights into the tissue-specific responses of *C. sapidus* to co-occurring legacy and emerging contaminants.

## 2. Materials and Methods

### 2.1. Crab Acclimation and Exposure

Specimens of *C. sapidus* (average carapace width of about 11 cm) were collected in a licensed area for bivalve culture in the Po Delta, near Porto Tolle (Rovigo, Italy), and promptly transported to the laboratory. Crabs were acclimated for one week in large aquaria containing seawater (temperature 12 °C, salinity 35), continuously aerated and fed ad libitum with *Mytilus galloprovincialis*. Only male crabs without obvious injuries or infection were used for the experiments.

Stock solutions (100 mg/L) of the tested contaminants were prepared by dissolving HgCl_2_ or BPS (Merck, Milano, Italy) in ultrapure distilled water. Eight tanks (30 L each) were then set up, each containing seven adult male specimens, according to the following scheme:

- Two control tanks containing only seawater;
- Two tanks with BPS at a concentration of 300 ng/L;
- Two tanks with Hg at a concentration of 300 ng/L;
- Two tanks with the mixture (MIX), containing 150 ng/L BPS and 150 ng/L Hg.

Every 48 h, the crabs were fed *M. galloprovincialis* (one mussel per crab). After feeding, the seawater was renewed totally, and the experimental concentrations of the two contaminants were restored in the tanks. *C. sapidus* specimens were exposed for 7 days, after which tissues were collected.

### 2.2. Tissue Collection and Preparation

Three specimens per tank were sampled, for a total of six animals per each experimental condition. hemolymph was collected from the abdominal segment by a 1 mL plastic syringe and stored in tubes on ice. After sampling, total hemocyte count (THC), hemocyte diameter and volume, lactate dehydrogenase (LDH) activity and hemocyte proliferation (XTT assay) were measured. The remaining hemolymph samples were centrifuged at 780× *g* for 10 min. Supernatant, namely cell-free hemolymph (CFH) was collected, whereas the pellets (=hemocytes) were resuspended in distilled water to obtain hemocyte lysate (HL). CFH and HL were then frozen in liquid nitrogen and stored at −80 °C until analyzed.

Gills and hepatopancreas were dissected from each crab. Each tissue was then divided into aliquots, frozen in liquid nitrogen, and stored at −80 °C until analysis. Before analyses, tissue supernatants (SN) were prepared by homogenizing gill or hepatopancreas samples in a homogenization buffer (10 mM Tris-HCl, pH 7.6 + 0.15 M KCl + 0.5 M sucrose + protease inhibitors) using a TissueLyser LT (Qiagen, Milano, Italy), then centrifuged at 12,000 rpm at 4 °C for 30 min.

### 2.3. Hemocyte Parameters

THC, hemocyte diameter and volume, were determined using a Scepter™ 2.0 Automated Cell Counter (MilliporeSigma, Burlington, MA, USA). Briefly, 20 μL of hemolymph were diluted into 2 mL of Coulter Isoton II diluent. The THC was expressed as the number of hemocytes (10^6^)/mL of hemolymph, while hemocyte diameter and volume were expressed in μm and picolitres (pL), respectively.

LDH activity in CFH (500 μL) was measured using a commercial kit (Cytotoxicity Detection Kit, Roche-Merck, Milano, Italy), following the manufacturer’s instructions. The results were expressed as optical density (OD) at 490 nm.

Hemocyte proliferation was evaluated using the Cell proliferation Kit II (Merck, Milano, Italy). A volume of 200 μL of the reagent mixture provided by the kit was added to 400 μL of pooled hemolymph and incubated for 4 h. The absorbance at 450 nm was then recorded and results were normalized to THC values of each experimental group and expressed as optical density (OD) at 450 nm.

The arylsulfatase activity was measured in both CFH and HL samples according to Zucker-Franklin et al. [33]. The amount of produced p-nitrocatechol was quantified after 1 h at 515 nm using a microplate reader and then calculated using the formula proposed by Baum et al. [34]. Results are expressed as μg of p-nitrocatechol produced per hour/mg of protein.

Both acid and alkaline phosphatases were also measured in HL and CFH. The acid phosphatase hydrolyses the substrate 4-nitrophenyl phosphate during the incubation at 37 °C and the absorbance was read at 405 nm using a microplate reader. Results were expressed as U/mg of protein. Similarly, the alkaline phosphatase hydrolyses the same substrate in an alkaline buffer and after the incubation at 30 °C the absorbance was recorded at 405 nm [35].

Phenoloxidase activity was quantified in both CFH and HL using L-DOPA (3,4-dihydroxy-L-phenylalanine, Sigma, Milano, Italy) as substrates. Briefly, 100 μL of HL or CFH were added to 900 μL of 1 mg L-DOPA/mL of phosphate-buffered saline and incubated for 30 min at 37 °C. Then, the absorbance at 490 nm was recorded and results were expressed as U/mg of proteins.

CFH hemocyanin concentration was measured at 335 nm using an extinction coefficient of 17.5 mmol L^−1^ cm^−1^, as proposed by Weber et al. [36] based on the A1%, 1 cm value of 2.33 reported for *Carcinus maenas* hemocyanin [37] and a functional-unit mass of 75 kDa. Results were expressed as mM of hemocyanin.

Glucose levels in CFH were determined using a coupled colorimetric reaction in which the glucose was oxidized to gluconic acid, producing hydrogen peroxide that reacts with o-dianisidine generating a colored compound that was quantified at 430 nm [38]. Results were expressed as mg glucose/mL of hemolymph.

### 2.4. Gill and Hepatopancreas Biomarkers

The CUPRAC (CUPric Reducing Antioxidant Capacity) method is a biochemical assay used to determine the total antioxidant capacity of a tissue. Following the protocol of Apak et al. [39], 50 µL of cupric chloride solution, 50 µL of acetate buffer, 50 µL of neocuproine, and 30 µL of SN were mixed. Absorbance was read at 450 nm, and results expressed as mM Trolox equivalents (vitamin E analogue)/mg protein.

Superoxide dismutase (SOD) activity was determined according to Crapo et al. [40]. The reaction mixture included phosphate buffer (50 mM, pH 8.6), hypoxanthine (13 mM in 1 N NaOH), cytochrome c (1.6 mM in phosphate buffer), SN and xanthine oxidase (20 U/mL in distilled water). Absorbance at 550 nm was monitored for 1 min. Results were expressed as U SOD/mg protein.

Catalase (CAT) activity was measured according to Aebi et al. [41] by monitoring absorbance decrease at 240 nm for 30 s using a mixture of substrate (phosphate buffer 50 mM + H_2_O_2_ 30%) and SN. Results were expressed as U CAT/mg protein.

Glutathione reductase (GR) activity was measured in both gills and hepatopancreas SN according to Smith et al. [42], by measuring the (5-thio (2-nitrobenzoic acid)) TNB production at 412 nm. The enzyme activity was expressed as U/mg protein.

Glutathione S-transferase (GST) activity was measured in hepatopancreas SN according to the method described in [43] using 1-chloro-2,4-dinitrobenzene (CDNB) and reduced glutathione (GSH) as substrates. GST activity was expressed as nmol/min/mg protein.

Electron transport system (ETS) was evaluated according to De Coen and Janssen [44]. SN samples were incubated with NADPH and NADH to saturate the electron transport system, then iodonitrotetrazolium (INT) was added as an electron acceptor, reduced to formazan (color shift from yellow to red/violet). Absorbance was measured at 490 nm using a Varioskan microplate reader (Life Technologies Italia, Segrate, Milano, Italy). Results were expressed as mol/min/mg protein.

Protein carbonyl content (PCC) was measured in duplicate using the method of Mecocci et al. [45] following the reaction with 2,4-dinitrophenylhydrazide (DNPH). Results were expressed as nmol carbonyl group/mg protein.

Lipid peroxidation (LPO) was quantified using the malondialdehyde (MDA) assay, according to the method of Buege and Aust [46]. Absorbance was read spectrophotometrically at 532 nm and the results were expressed as nmoles of thiobarbituric reactive substances (TBARS)/mg protein. TBARS, considered as “MDA-like peroxide products”, were quantified by reference to MDA absorbance (ε = 156 × 10^3^ M^−1^ cm^−1^) [47].

Acetylcholinesterase (AChE) activity was measured in gills following the colorimetric reaction between acetylthiocholine and the reagent dithiobisnitrobenzoate [48]. Absorbance was recorded at 405 nm for 5 min on a microplate reader at room temperature. The results were expressed as nmol/min/mg of protein. Butyrylcholinesterase (BChE) activity was measured according to Escartín and Porte [49] using butyrylthiocholine as the substrate. Results are expressed as nmol/min/mg protein.

Total protein content of each SN sample was determined using the Bradford method [50].

### 2.5. Transmission Electron Microscopy (TEM)

Hemocytes, gills and hepatopancreas were used for TEM observation. hemocytes, gills and hepatopancreas were fixed for 1 h (ON for tissues) at 4 °C in 2.5% glutaraldehyde in sodium cacodylate buffer (0.2 M, pH 7.2) plus 1.7% NaCl and 1% sucrose. Samples were postfixed with 1% osmium tetroxide plus potassium ferrocyanide 1% in 0.1 M sodium cacodylate buffer for 1 h at 4 °C. After three water washes, samples were dehydrated in a graded ethanol series, infiltrated with propylene oxide and embedded in epoxy resin (Sigma-Aldrich, Milano, Italy). Ultrathin sections (100 nm) were obtained with a Leica Ultracut EM UC7 (Leica Biosystems Nussloch Gmb, Nußloch, Germany) ultramicrotome equipped with a Diatome diamond knife, they were stained with saturated uranyl acetate in 50% ethanol followed by 1% lead citrate and finally observed with a Tecnai G2 (FEI) transmission electron microscope operating at 100 kV. Images were captured with an Olympus (OSIS) Veleta digital camera.

### 2.6. Statistical Analysis

Statistical analysis was performed using the software OriginPro (Version 2025. OriginLab Corporation, Northampton, MA, USA). Data were checked for normal distribution (Shapiro–Wilk’s test) and homogeneity of variances (Bartlett’s test). Results were compared by a one-way ANOVA, followed by Fisher’s post hoc test for pairwise comparison. Results were represented as boxplots. Statistically significant difference was set at *p* < 0.05. Principal Component Analysis (PCA) of the results obtained in each tissue, was also performed.

## 3. Results

### 3.1. Hemocyte Parameters

**Figure 1:**
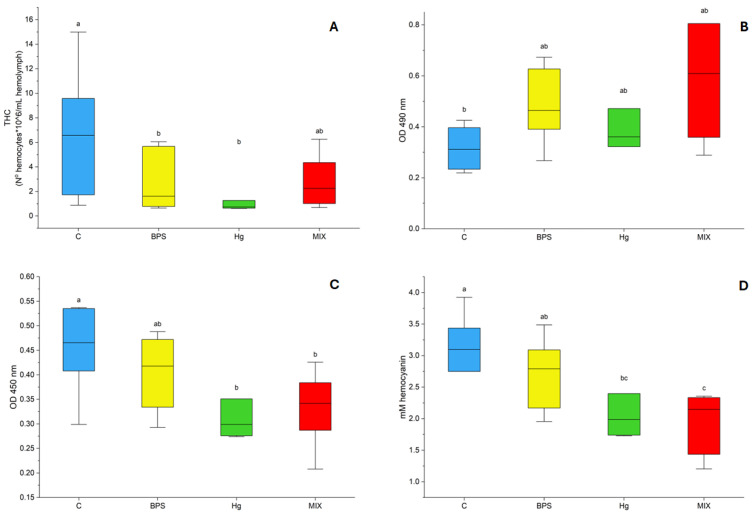
Boxplots of total hemocyte count (THC), expressed as n° hemocytes (10^6^)/mL hemolymph (**A**), LDH activity, expressed as OD 490 nm (**B**), hemocyte proliferation, expressed as OD 450 nm (**C**), and hemocyanin, expressed as mM (**D**) results. Different letters indicate significant differences among experimental conditions. N = 6.

Statistical analysis demonstrated that Hg and BPS, but not MIX, reduced (*p* < 0.05) THC values significantly after 7 days of exposure, when compared to the control group (Figure 1A), whereas exposure to tested contaminants did not affect hemocyte volume and diameter.

Experimental conditions did not induce statistically significant cytotoxic effects (LDH activity), even if LDH activity generally increased in Hg-, BPS- and MIX-treated crabs, with respect to the controls (Figure 1B).

Exposure to Hg and MIX reduced (*p* < 0.05) hemocyte proliferation significantly (Figure 1C), with respect to the control.

Moreover, the post hoc test revealed a significant (*p* < 0.05) decrease in hemocyanin levels in the hemolymph of crabs exposed to Hg and MIX (Figure 1D).

**Figure 2:**
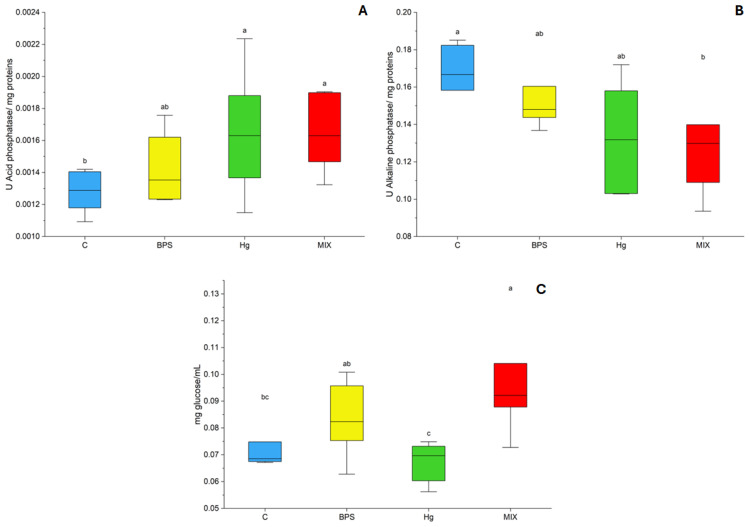
Boxplots of acid phosphatase activity in CFH, expressed as U/mg protein (**A**), alkaline phosphatase activity in CFH, expressed as U/mg protein (**B**), and glucose levels, expressed as mg/mL hemolymph (**C**). Different letters indicate significant differences among experimental conditions. N = 6.

As for hydrolytic enzymes, alkaline and acid phosphatase, as well as arylsulfatase activities were not affected in HL. Conversely, the exposure of crabs to Hg and MIX increased (*p* < 0.05) acid phosphatase significantly in CFH (Figure 2A), while only MIX induced a significant (*p* < 0.05) reduction in CFH alkaline phosphatase activity (Figure 2B). No significant changes were observed in CFH arylsulfatase activity.

Exposure of crabs to MIX caused a significant increase in CFH glucose levels (*p* < 0.05) (Figure 2C), whereas none of the experimental conditions induced significant variations in PO activity, either in HL or in CFH.

### 3.2. Gill and Hepatopancreas Biomarkers

**Figure 3:**
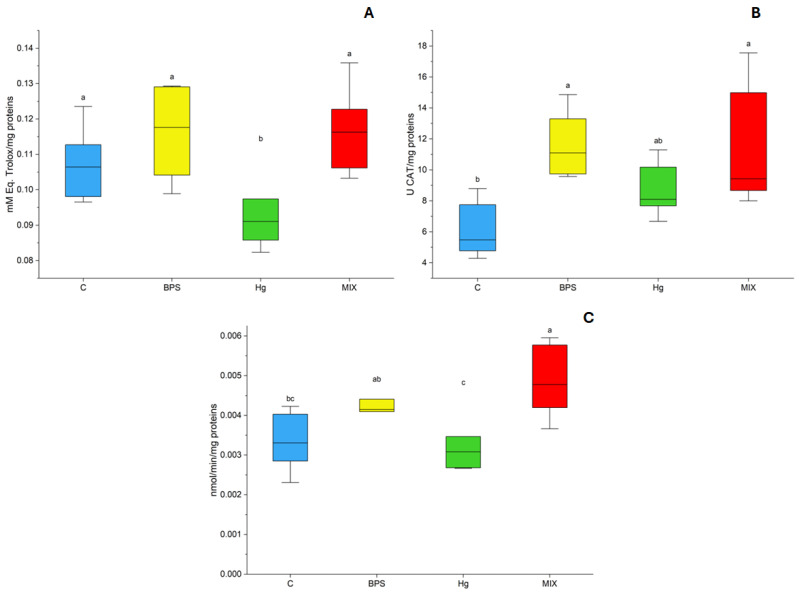
Boxplots of gill total antioxidant capacity, expressed as mM of Trolox equivalents/mg proteins (**A**), CAT activity, expressed as U/mg proteins (**B**) and ETS, expressed as nmol/min/mg proteins (**C**). Different letters indicate significant differences among experimental conditions. N = 6.

Statistical analysis (one-way ANOVA) revealed a significant effect of treatment on total antioxidant capacity (CUPRAC) in gills (*p* < 0.01). In particular, the post hoc Fisher’s test indicated that exposure to Hg led to a significant decrease in CUPRAC values, compared with the control group (Figure 3A). In hepatopancreas, no significant differences in CUPRAC values were observed among treatments.

ANOVA showed no significant differences in SOD activity among treatments (*p* > 0.05) both in gills and hepatopancreas, whereas exposure to BPS and MIX induced significant increases in CAT activity only in gills (Figure 3B).

GR activity was not significantly influenced by any treatment in both the tissues, as well as GST activity in hepatopancreas.

ETS activity in gills was significantly influenced by treatment (*p* < 0.01). Post hoc analysis showed that crabs exposed to the MIX exhibited a significant increase in ETS activity compared with controls, while no significant variations were observed in the groups exposed to BPS or mercury alone (Figure 3C). Conversely, no significant effects were observed in the ETS of hepatopancreas.

**Figure 4:**
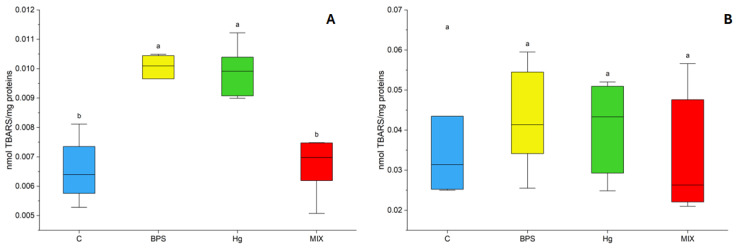
Boxplots of LPO in gills (**A**) and hepatopancreas (**B**), expressed as noml TBARS/mg proteins. Different letters indicate significant differences among experimental conditions. N = 6.

A statistically significant effect of exposure to the contaminants tested was detected in gill LPO levels (*p* < 0.001). Gills from crabs exposed to either BPS or mercury displayed significantly higher LPO, when compared to controls (Figure 4A). Although a slight increase in LPO levels was observed in the hepatopancreas of crabs exposed to the experimental conditions, the increase was not statistically significant (Figure 4B) with respect to controls.

In gills, both AChE and BChE activities did not change significantly after 7 days of exposures of crabs to Hg, BPS and MIX.

### 3.3. TEM Analysis

**Figure 5:**
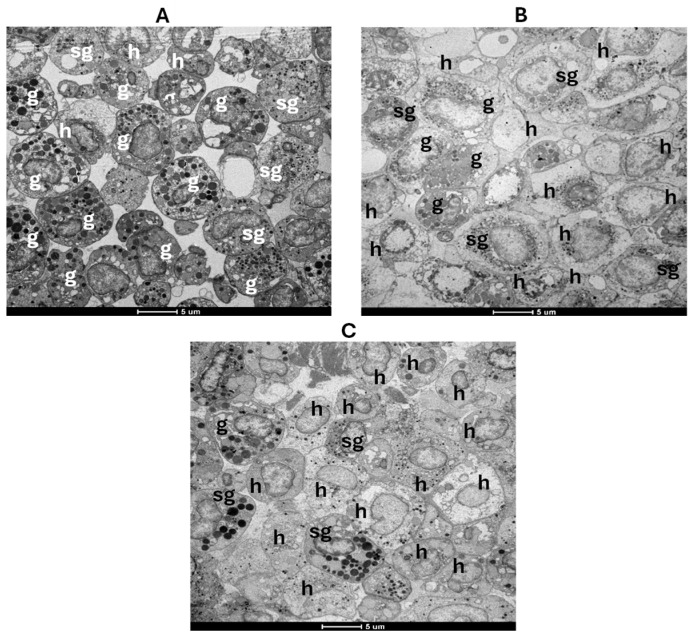
TEM micrographs of *C. sapidus* hemocytes from control (**A**), BPS-treated (**B**) and MIX-treated (**C**) groups. **g**: granulocytes; **sg**: semigranulocytes; **h**: hyalinocytes. Bar length: 5 μm.

Observations under TEM confirmed the presence of three hemocyte types in the hemolymph of *C. sapidus*, namely granulocytes (with numerous large electron-dense granules), semigranulocytes (with fewer granules than granulocytes), and hyalinocytes (with absent or sparse granules). Interestingly, the fraction of granulocytes (particularly high in controls) tended to decrease under exposure conditions, particularly in crabs exposed to BPS and MIX. In the latter two cases, the frequency of semigranulocytes and hyalinocytes increased (Figure 5A–C).

**Figure 6:**
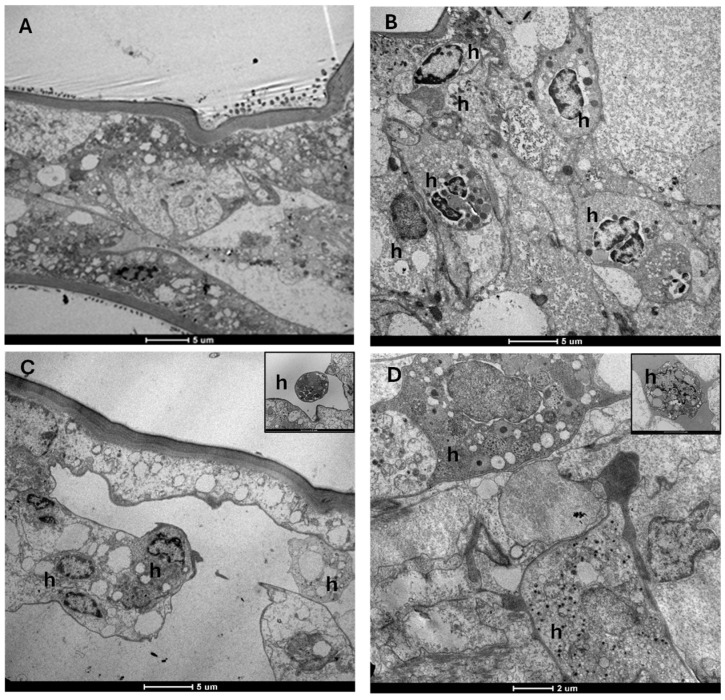
TEM micrographs of *C. sapidus* gills from control (**A**), BPS-treated (**B**), Hg-treated (**C**) and MIX-treated (**D**) groups. **h**: hemocytes. Bar length: 5 μm for (**A**–**C**); 2 μm for (**D**) and inserts.

In gills, no alterations in the tissue structure were highlighted, but rather an evident infiltration of hemocytes was noted inside the gill tissue of crabs exposed to BPS, Hg and MIX (Figure 6A–D).

**Figure 7:**
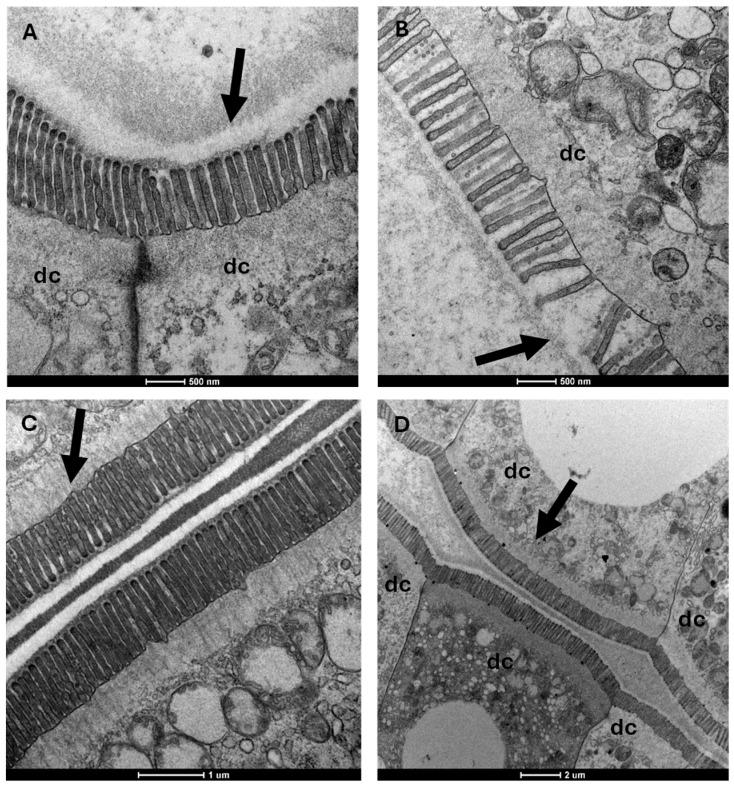
TEM micrographs of *C. sapidus* hepatopancreas from control (**A**), BPS-treated (**B**), Hg-treated (**C**) and MIX-treated (**D**) groups. **dc**: digestive cell. Arrows: microvilli. Bar length: 500 nm for (**A**,**B**), 1 μm for C and 2 μm for (**D**).

In the hepatopancreas, no structural alterations were observed in the digestive cells of treated crabs compared to those of the controls. Digestive cells appeared as columnar cells with a well-developed brush border of microvilli. Microvilli were well arranged under almost all experimental conditions, except in the hepatopancreas of crabs exposed to BPS, in which portions of disorganized microvilli were detected (Figure 7A–D).

### 3.4. PCA Analyses

The PCA analyses provided an integrated overview of the biomarker responses measured in hemolymph, gills, and hepatopancreas of *C. sapidus* exposed to Hg, BPS, and MIX.

In hemolymph (Figure S4), the first principal component (PC1) explained 27.45% of the total variance, whereas PC2 explained 16.38%, accounting together for 50.60% of the overall variability. The PCA showed a partial separation of controls from exposed crabs along PC1. Control specimens tended to cluster on the positive side of PC1, associated mainly with THC, hemocyanin, hemocyte diameter and volume, and CFH enzymatic activities, whereas Hg-exposed crabs were mainly distributed on the negative side of PC1, reflecting reductions in hemocyte parameters. BPS- and MIX-exposed crabs showed an intermediate distribution, partially overlapping with controls, suggesting a less pronounced but still detectable alteration of hemolymph responses. Variables such as LDH activity, glucose levels, and phosphatase activities contributed mainly to the positive side of PC2, indicating a relationship with stress-related and lysosomal responses.

In gills (Figure S5), PC1 explained 31.07% of the variance and PC2 explained 22.09%, together accounting for 53.15% of the total variance. A clearer separation among treatments was observed in gills compared to hemolymph. Hg-exposed crabs were mainly distributed on the negative side of PC1 and were associated with increased LPO, supporting the occurrence of oxidative damage. Conversely, BPS- and MIX-exposed crabs were generally located on the positive side of PC1, where CAT, ETS, BChE, and AChE vectors were oriented, indicating enhanced metabolic and antioxidant responses. Control samples occupied an intermediate position, suggesting a physiological baseline condition. Overall, the PCA confirms that gills were among the most responsive crab tissues to contaminant exposure, particularly in terms of oxidative stress.

In the hepatopancreas (Figure S6), PC1 accounted for 37.87% of the total variance, while PC2 explained 21.58%, for a cumulative variance of 60.40%. Unlike hemolymph and gills, the hepatopancreas PCA showed a substantial overlap among experimental groups, indicating limited biochemical differentiation between control and exposed crabs. Nevertheless, some Hg- and MIX-exposed specimens were associated with CAT and ETS activity, whereas BPS-exposed crabs showed a partial association with LPO. Control organisms were generally positioned closer to GR and GST vectors, suggesting maintenance of antioxidant and detoxification homeostasis. The broad overlap among groups supports the hypothesis that the hepatopancreas was more resistant to short-term exposure than the other investigated tissues.

## 4. Discussion

The present study provides new insights into the tissue-specific effects of mercury and BPS, alone and in combination, in *C. sapidus*, highlighting differential sensitivity among hemolymph, gills, and hepatopancreas, and revealing complex, non-additive interactions between legacy and emerging contaminants.

### 4.1. Hemocyte Parameters

The significant reduction in THC following Hg and BPS exposure indicates a clear cellular effect of the two contaminants. In marine invertebrates, similar decreases in circulating hemocytes have been widely reported in organisms exposed to heavy metals, including Hg, Cd, and Cu, and are generally attributed to cell death (apoptosis), impaired hematopoiesis, or migration of hemocytes toward damaged tissues [51,52]. In crabs in particular, exposure for 7 and 14 days to 10 μg/L of inorganic mercury reduced significantly THC values in the crab *Scylla serrata* [53]. After 96 h of exposure to concentrations higher than 50 μg/L of mercury (as HgCl_2_) induced a significant reduction in THC values also in the freshwater prawn *Macrobrachium rosenbergii*, when compared to the control groups [54]. In the present study, BPS also caused a significant reduction in THC values in crabs. Conversely, a recent study demonstrated that BPS was unable to induce significant alterations in THC values in the crab *Carcinus aestuarii* [55]. This suggests that the effects of BPS in crabs can be species-specific. In this study, the observed inhibition of hemocyte proliferation mostly in Hg- and MIX-exposed crabs suggested that animals were unable to mitigate the reduction in circulating hemocyte number. From a mechanistic point of view, the infiltration of hemocytes observed in the gills of *C. sapidus* exposed to Hg and MIX suggests a migration of hemocytes towards peripheral tissues and that cellular proliferation could not compensate, at least partially, for the reduction in THC. In addition, hemocytes from *C. sapidus* did not show significant alterations in both diameter and volume after the exposure to Hg, BPS and MIX, suggesting that the experimental conditions tested did not affect cell morphology, as revealed by the Scepter™ 2.0 Automated Cell Counter analysis. However, in this study, TEM observations indicated a gradual reduction in cell granularity in treated crabs when compared to control groups, suggesting that experimental conditions tested (mainly Hg and MIX) were able to provoke cell degranulation due to the reduction in cell membrane stability. In this regard, Singaram et al. [53] demonstrated that Hg can significantly reduce cell membrane stability in hemocytes from *S. serrata* exposed for 7 and 14 days to sublethal concentrations of mercury. hemocyte degranulation was observed in other crab species exposed to contaminants [56,57] or immune stimuli [58]. In this study, the hypothesis of a degranulation process suffered by hemocytes from treated crabs can be supported by the increase (although not in a statistically significant manner) of LDH activity in the hemolymph at all the experimental conditions tested. LDH is a stable, cytoplasmic enzyme found in almost all cells. It is widely used as a biomarker for assessing cytotoxicity and cell damage because it is released into the extracellular environment—such as cell culture supernatant, hemolymph, or blood—when the plasma membrane is damaged [59].

In the present survey, the enzymatic alterations observed in CFH (increase in acid phosphatase activity in Hg- and Mix-exposed crabs and decrease in alkaline phosphatase activity in Mix-exposed crabs) suggested lysosomal membrane destabilization and activation of degradative pathways. This is consistent with contaminant-induced lysosomal perturbation, a well-established biomarker of cellular stress in marine invertebrates [60]. Conversely, in this study BPS alone did not affect hydrolytic enzyme activities markedly in both the CFH and HL of *C. sapidus*. Likewise, HL lysozyme activity, as well as arylsulfatase and acid phosphatase activities, were not affected in both HL and CFH following exposure of the crab *C. aestuarii* to bisphenols [55].

The decrease in hemocyanin levels observed in the present study in Hg- and MIX-exposed animals is particularly noteworthy, as this protein plays a crucial role in oxygen transport and immune defense in crabs [61,62]. We hypothesized a direct interaction between Hg (also present in the MIX) and the hemocyanin of *C. sapidus* hemocytes. Interestingly, Brouwer et al. [61] demonstrated that *C. sapidus* hemocyanin possesses at least three mercury binding sites. All this evidence suggests that Hg can interfere (e.g., competition for binding sites) with the binding of oxygen to *C. sapidus* hemocyanin.

In this study, the measurement of CFH glucose levels was used as a biomarker of stress in crabs exposed to Hg and BPS. Indeed, previous studies demonstrated that glucose levels can increase in hemolymph from crabs exposed to differing stressful conditions [63,64,65,66,67], including heavy metals [68,69]. The hyperglycemic response in MIX-exposed crabs observed in this study indicates an induction of metabolic stress, as also suggested by Qyli et al. [69] in the crab *C. maenas* following exposure to various stressors, such as copper, hypoxia, temperature, chloroform and adrenaline.

### 4.2. Gill and Hepatopancreas Biomarkers

Gills of *C. sapidus* exhibited the most pronounced biochemical responses to the experimental conditions tested, confirming their role as a primary target tissue for waterborne contaminants. The decrease in total antioxidant capacity (CUPRAC) following Hg exposure reflects depletion of the general antioxidant capability of such tissue. Interestingly, BPS did not induce significant alterations in CUPRAC levels neither in gills nor in hepatopancreas of *C. sapidus*. Conversely, a recent study demonstrated that BPS was able to decrease the total antioxidant capacity of gills after 7 days of exposure of the crab *C. aestuarii* [55], suggesting a species-specific mechanism of action of this contaminant. In this study, no significant variations in SOD activity were recorded in gills and hepatopancreas after exposure of *C. sapidus* to Hg, BPS and MIX, suggesting that superoxide radical production may not be the primary driver of oxidative imbalance in this case, or that SOD activity was already operating near maximal capacity. Instead, the increase in CAT activity observed in gills from BPS- and MIX-treated crabs suggested a compensatory response to elevated hydrogen peroxide levels, under the experimental conditions tested at least. Similarly, exposure for 7 days to BPS induced a marked—even if not significant—increase in CAT activity in hepatopancreas of *C. aestuarii* [55]. In both the gills and hepatopancreas of S. serrata, exposure for 7 days to Hg induced a significant decrease in SOD activity only at a concentration of 10 μg/L, whereas a prolonged exposure (14 days) significantly decreased enzyme activity at 1 μg/L [53]. In the latter study, gill CAT activity showed a similar pattern of variation in SOD, whereas a significant reduction in CAT activity was observed in the hepatopancreas of crabs exposed to both Hg concentrations after 7 days of exposure [53]. In the hepatopancreas of *Eriocheir sinensis*, SOD and CAT activity of crabs exposed for 40 days to 0.01 and 0.05 mg/L Hg^2+^ were significantly higher than in the controls, whereas enzyme activities decreased significantly in crabs exposed to 0.1, 0.2 and 0.3 mg/L, suggesting a biphasic mechanism of action of mercury depending on its exposure concentrations [70].

The significant increase in LPO levels in gills from Hg- and BPS-exposed crabs confirms that oxidative stress translates into oxidative damage at the membrane level, probably owing to an imbalance between pro-oxidant and antioxidant processes. Interestingly, the absence of a significant LPO increase in the MIX group suggests antagonistic or compensatory interactions between BPS and Hg.

In this study, the increase in ETS activity observed in the gills of MIX-exposed crabs indicates an enhanced metabolic demand in this tissue. A recent study evaluated the effects of a BPA analogue-contaminated diet in *C. aestuarii* specimens reporting no alterations in their ETS activity, further suggesting a species-specific response [71]. Regarding the effects of mercury on ETS activity of crustaceans, to the best of our knowledge, no studies have been conducted so far.

As for AChE e BChE activity, the present study demonstrated that Hg, BPS and MIX did not affect enzyme activities in crab gills, suggesting the absence of neurotoxic effects of the experimental conditions tested. This is a surprising result, at least for Hg because literature data indicate that mercury can inhibit AChE activity in marine organisms, including crabs. In particular, exposure for 24 and 48 h of crayfish, *Procambarus clarkii*, to 0.2 mg/L of mercuric chloride resulted in a significant decrease in AChE activity [72]. Exposure for 96 h to Hg induced a significant decrease in AChE activity also in the crab *C. maenas* at all the concentrations tested (0.09, 0.19, 0.37 and 0.74 mg/L) [73]. These findings indicate that Hg can interfere with neurotransmission in cholinergic synapses of crabs. However, it is important to note that the concentrations (in the order of hundreds of micrograms/L) and exposure duration (max 96 h) applied in the cited studies are different from those of the present study, in which crabs were exposed to lower concentrations of Hg (300 ng/L) and for longer periods (7 days) than in the aforementioned studies. Therefore, we cannot exclude that in our study, environmentally relevant Hg concentrations were not able to induce a reduction in enzymatic activities. Alternatively, the prolonged 7-day exposure of *C. sapidus* could have allowed the crabs to acclimate to the experimental conditions, resulting in the absence of neurotoxicity of the tested contaminants.

Overall, in contrast to gills, crab hepatopancreas showed limited biochemical alterations, suggesting effective detoxification and antioxidant defense during short-term exposure. This organ plays a central role in xenobiotic metabolism [74]. In this study, the lack of significant changes in antioxidant enzymes (SOD, CAT, GR, GST) and LPO suggests that the hepatopancreas of *C. sapidus* successfully maintained redox homeostasis. Our findings also suggest that hepatopancreatic responses can require longer exposure times or higher contaminant concentrations to become evident. However, it is important to note that the absence of biochemical changes does not exclude subcellular or ultrastructural damage, which may precede measurable enzymatic responses, as observed in TEM analysis (see below).

### 4.3. TEM Observations

The ultrastructural observations obtained through TEM provide important complementary evidence to the biochemical and cellular responses, allowing a deeper understanding of the mechanisms underlying contaminant toxicity in *C. sapidus*. In particular, changes observed in hemocytes, gills, and hepatopancreas highlight both adaptive and stress-related responses at the subcellular level.

In hemocytes, the reduction in the relative abundance of granulocytes, accompanied by an apparent increase in semigranulocytes and hyalinocytes, suggests a shift in hemocyte population dynamics under contaminant exposure. Granulocytes are generally considered one of the main effector cell types in crustacean immune responses, being rich in cytoplasmic granules containing hydrolytic enzymes and antimicrobial compounds [75]. Their decrease, together with the observed reduction in granularity, is indicative of degranulation processes. Similar ultrastructural alterations have been reported in marine organisms exposed to heavy metals and organic pollutants, where degranulation is associated with the release of lysosomal contents and immune mediators [51,52].

In this study, TEM evidence of hemocyte infiltration into gill tissues strongly supports the hypothesis of hemocyte migration from the hemolymph to peripheral tissues, likely in response to local damage or inflammation. Gills are a primary interface with the external environment and are particularly susceptible to waterborne contaminants. The presence of hemocytes inside gill tissue may therefore explain, at least in part, the reduction in THC measured in the hemolymph of *C. sapidus* exposed to BPS and Hg. Our findings also suggest that early stages of contaminant-induced damage in gills can be characterized by immune cell recruitment rather than immediate tissue disruption. Therefore, the lack of visible ultrastructural damage does not exclude functional impairment, especially considering the oxidative stress responses observed at the biochemical levels. This highlights the sensitivity of TEM in detecting early or subtle alterations that precede more severe histopathological changes.

In the hepatopancreas, the general preservation of cellular architecture, including well-developed microvilli and normal organization of digestive cells, confirms the relative resilience of this organ to short-term exposure. TEM observations revealed that R-cells (resorptive) were the most abundant cell types in the tubule epithelium of adults of *C. sapidus*, as previously described in other crab species [76]. However, localized disorganization of microvilli was observed in BPS-exposed crabs, suggesting an early sign of subcellular stress affecting absorptive and digestive functions.

Overall, TEM findings reinforce the concept that ultrastructural changes can occur even when biochemical responses are limited or absent, particularly in organs with high detoxification capacity, such as the hepatopancreas. In addition, the combined exposure to BPS and Hg (MIX) did not produce more severe ultrastructural damage compared to single treatments, supporting the evidence of non-additive (potentially antagonistic) interactions between mercury and BPS.

## 5. Conclusions

This study demonstrates that short-term exposure to mercury and bisphenol S induces significant, tissue-specific biological responses in *C. sapidus*, with clear implications for ecotoxicological assessment in coastal environments. Among the investigated tissues, gills proved to be the most sensitive, exhibiting pronounced oxidative stress, as evidenced by decreased antioxidant capacity and increased lipid peroxidation. These responses highlight the vulnerability of this tissue as a primary interface with the external environment and support its use as an early-warning indicator of contaminant exposure. In contrast, the hepatopancreas displayed a higher resistance to short-term exposure, likely due to its central role in detoxification and the efficiency of its antioxidant and biotransformation systems. At the systemic level, hemolymph biomarkers revealed clear signs of immunotoxicity, including reduced hemocyte counts, impaired proliferation, and alterations in enzyme activities, suggesting that both contaminants can compromise immune competence even at environmentally relevant concentrations.

TEM analysis revealed that the primary effects of Hg and BPS at the subcellular level involve hemocyte activation and degranulation, immune cell migration, and early structural alterations in target tissues, providing mechanistic support to the physiological and biochemical responses observed. These findings underline the importance of integrating ultrastructural approaches in ecotoxicological studies, as they allow the detection of early warning signals of stress that may not yet be reflected in conventional biomarkers.

Several biomarkers showed attenuated or distinct responses compared to single-compound treatments, indicating complex interactions between legacy and emerging contaminants. These findings underscore the limitations of single-compound approaches in predicting ecological risk and highlight the need to incorporate mixture toxicity into environmental risk assessment frameworks.

Overall, this study contributes to the growing evidence that bisphenol analogues, such as BPS, are biologically active contaminants capable of inducing measurable physiological and cellular responses in marine organisms. Given the increasing co-occurrence of such contaminants in marine ecosystems, further research should focus on long-term exposures, trophic transfer, and multi-stressor scenarios to better understand their ecological consequences.

As a final remark, we are aware that a limitation of the present study is the absence of quantification of Hg and BPS bioaccumulation. The experimental design was primarily focused on biomarker and ultrastructural responses, and tissue availability was largely allocated to biochemical analyses. Future studies should integrate chemical quantification of contaminant burdens to better correlate bioaccumulation patterns with biological responses.

## Data Availability

The original contributions presented in this study are included in the article/Supplementary Materials. Further inquiries can be directed to the corresponding authors.

## Supplementary Materials

The following supporting information can be downloaded at: https://www.mdpi.com/article/10.3390/jox16030096/s1, Figure S1: original TEM micrographs of *C. sapidus* hemocytes. Figure S2: original TEM micrographs of *C. sapidus* gills. Figure S3: original TEM micrographs of *C. sapidus* hepatopancreas. Figure S4: PCA analysis of results obtained in hemolymph from crabs exposed to Hg, BPS and MIX; Figure S5: PCA analysis of results obtained in gills from crabs exposed to Hg, BPS and MIX; Figure S6: PCA analysis of results obtained in hepatopancreas from crabs exposed to Hg, BPS and MIX.

## Abbreviations

The following abbreviations are used in this manuscript:

BPS	Bisphenol S
BPA	Bisphenol A
MIX	Mixture
TEM	Transmission Electron Microscopy
Hg	Mercury
THg	Total Mercury
THC	Total Hemocyte Count
LDH	Lactate Dehydrogenase
CFH	Cell-free Hemolymph
HL	Hemocyte Lysate
SN	Supernatant
OD	Optical Density
L-DOPA	3,4-dihydroxy-L-phenylalanine
PO	Phenoloxidase
CUPRAC	Cupric Reducing Antioxidant Capacity
SOD	Superoxide Dismutase
CAT	Catalase
GR	Glutathione Reductase
GST	Glutathione S-transferase
ETS	Electron Transport System
PCC	Protein carbonyl content
LPO	Lipid Peroxidation
MDA	Malondialdehyde
TBARS	Thiobarbituric Reactive Substances
AChE	Acetylcholinesterase
BChE	Butyrylcholinesterase
