# Supplementary material for: Individual and Combined Effects of Legacy and Emerging Contaminants on the Blue Crab Callinectes sapidus: Mercury and Bisphenol S as a Case Study
Source: J Xenobiot. 2026 May 29;16(3):96. doi: 10.3390/jox16030096 (PMC13302245; doi:10.3390/jox16030096)

Supplementary Materials: Individual and Combined Effects of Legacy and Emerging Contaminants on the Blue Crab *Callinectes sapidus*: Mercury and Bisphenol S as a Case Study

Jacopo Fabrello, Giovanni Martino Rigodanza, Francesco Boldrin, Federico Caicci, Marco Munari and Valerio Matozzo

Figure S1. Original TEM micrographs of *C. sapidus* hemocytes. From control (**A**), BPS-treated (**B**) and MIX-treated (**C**) groups.

Figure S2. Original TEM micrographs of *C. sapidus* gills. From control (**A**), BPS-tretaed (**B**), Hg-treated (**C**) and MIX-treated (**D**) groups.

Figure S3. Original TEM micrographs of *C. sapidus* hepatopancreas. From control (**A**), BPS-treated (**B**), Hg-treated (**C**) and MIX-treated (**D**) groups.

Figure S4. PCA analysis of results obtained in hemolymph from crabs exposed to Hg, BPS and MIX.

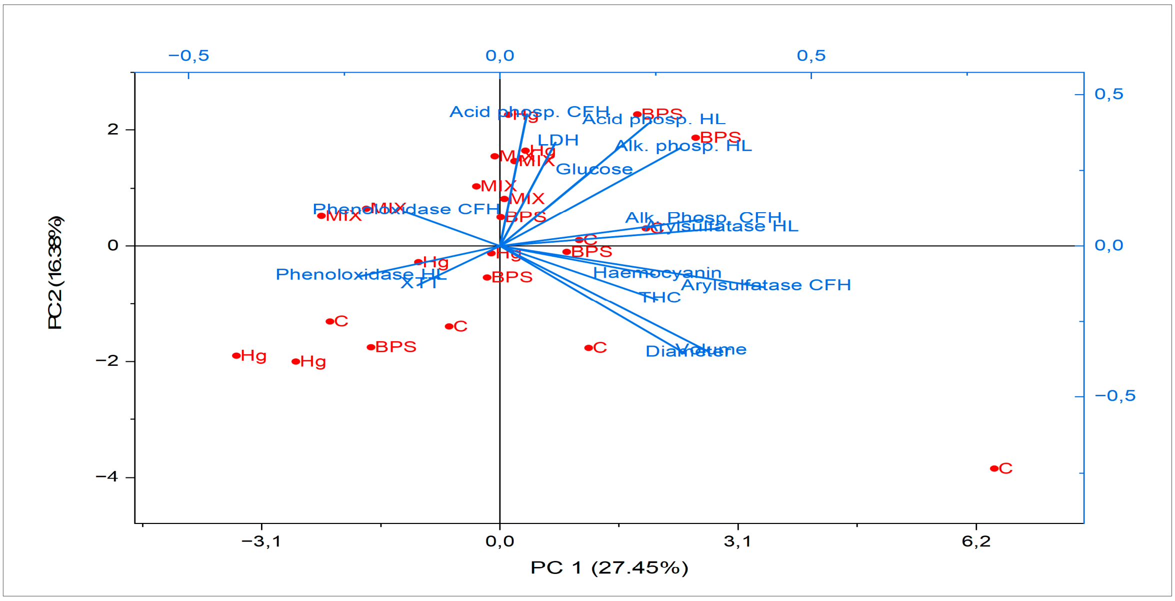

Figure S5. PCA analysis of results obtained in gills from crabs exposed to Hg, BPS and MIX.

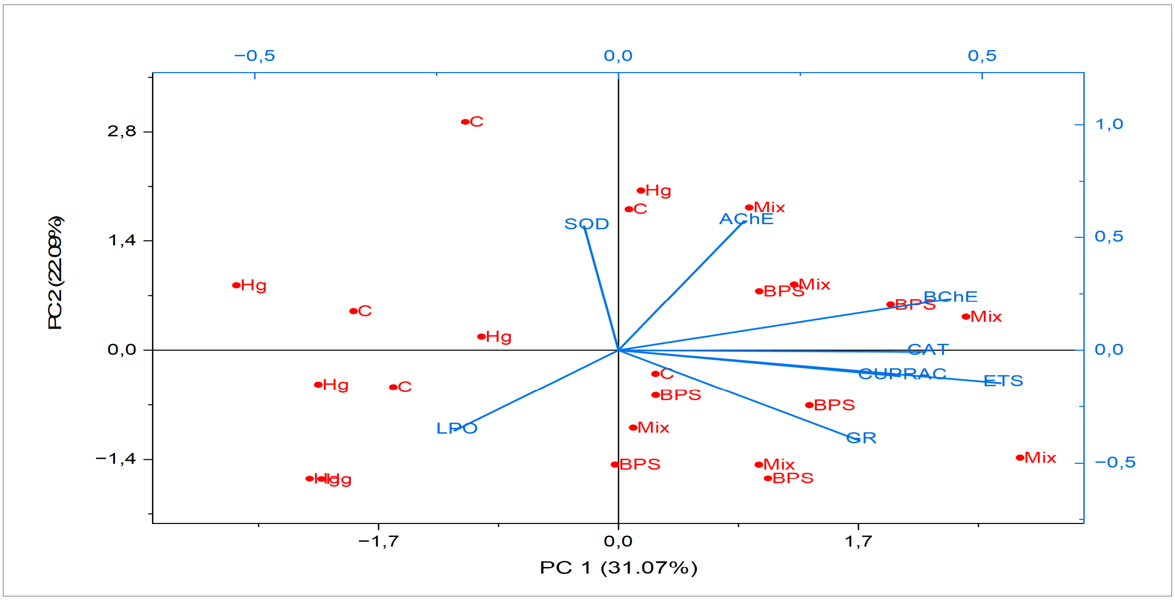

Figure S6. PCA analysis of results obtained in hepatopancreas from crabs exposed to Hg, BPS and MIX.

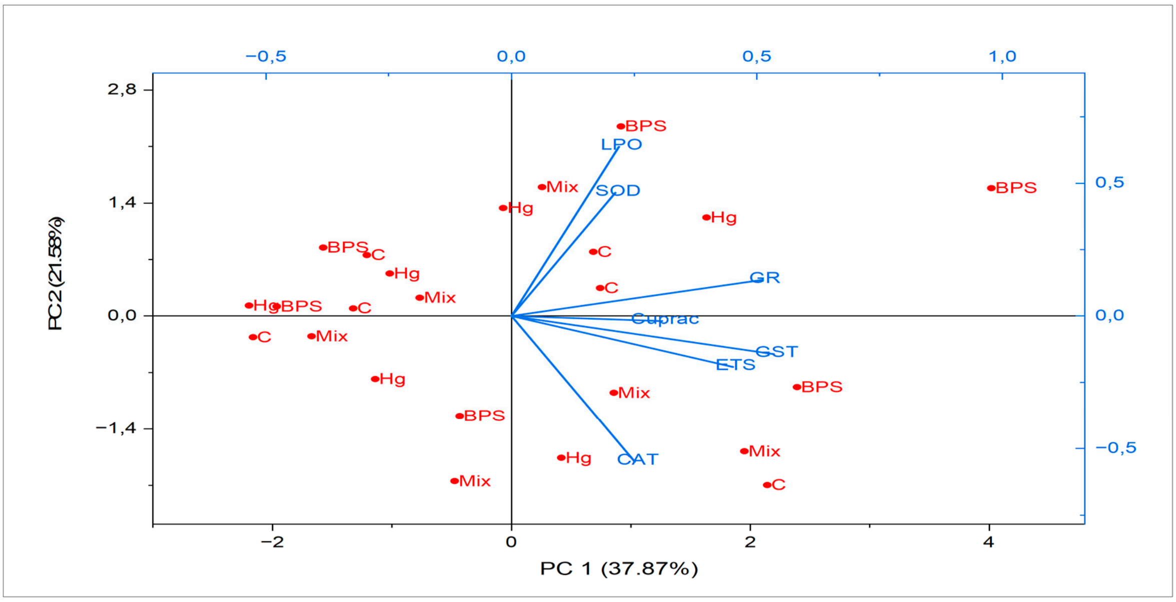

Supplement: Supplementary file 1 [file jox-16-00096-s001.zip › jox-4325455-supplementary.pdf]
