# Supplementary figures and images for: Individual and Combined Effects of Legacy and Emerging Contaminants on the Blue Crab Callinectes sapidus: Mercury and Bisphenol S as a Case Study
Source: J Xenobiot. 2026 May 29;16(3):96. doi: 10.3390/jox16030096 (PMC13302245; doi:10.3390/jox16030096)

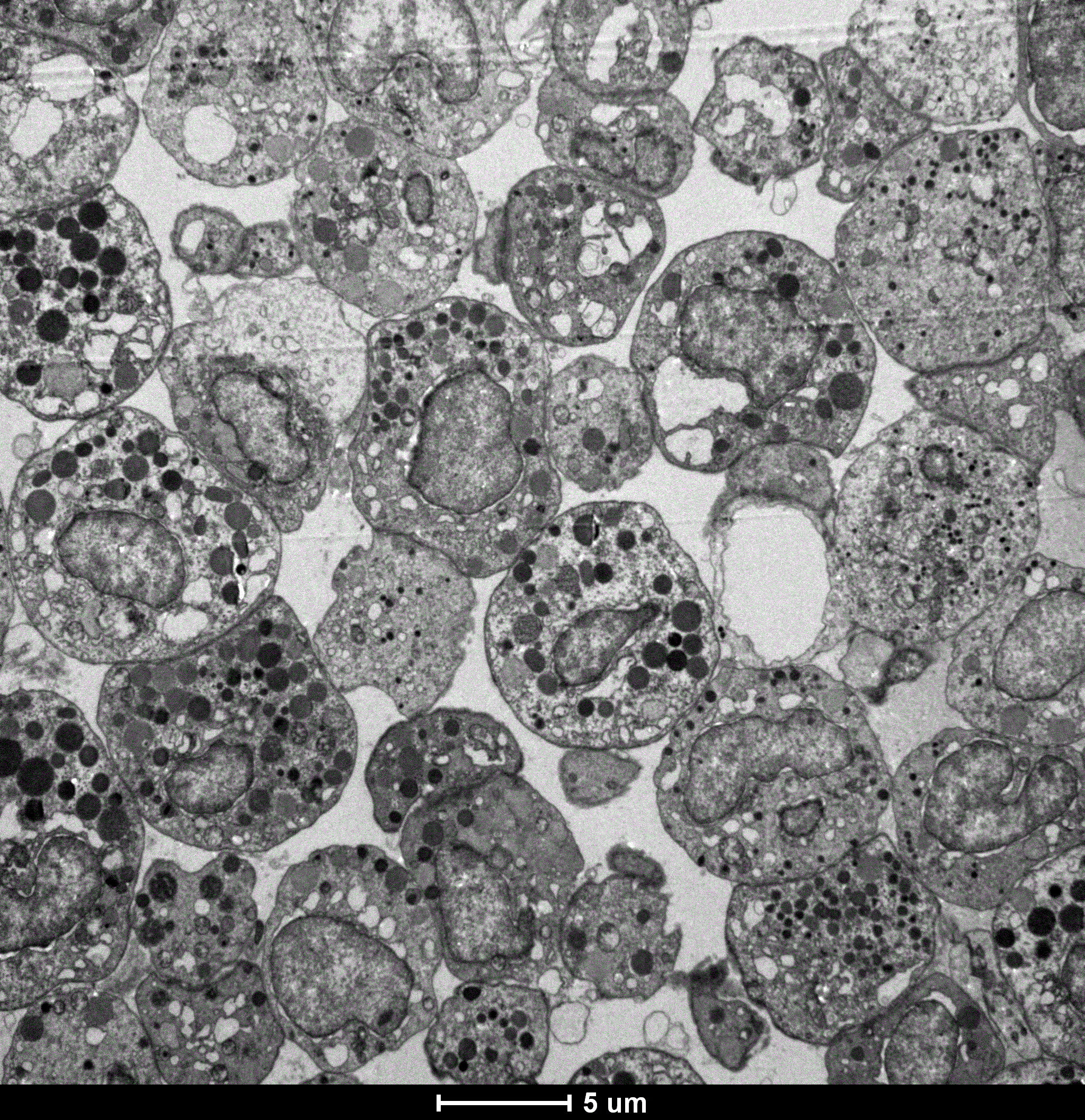

Supplement: Supplementary file 1 [file jox-16-00096-s001.zip › FigureS1-S3/Figure S1A.gif]

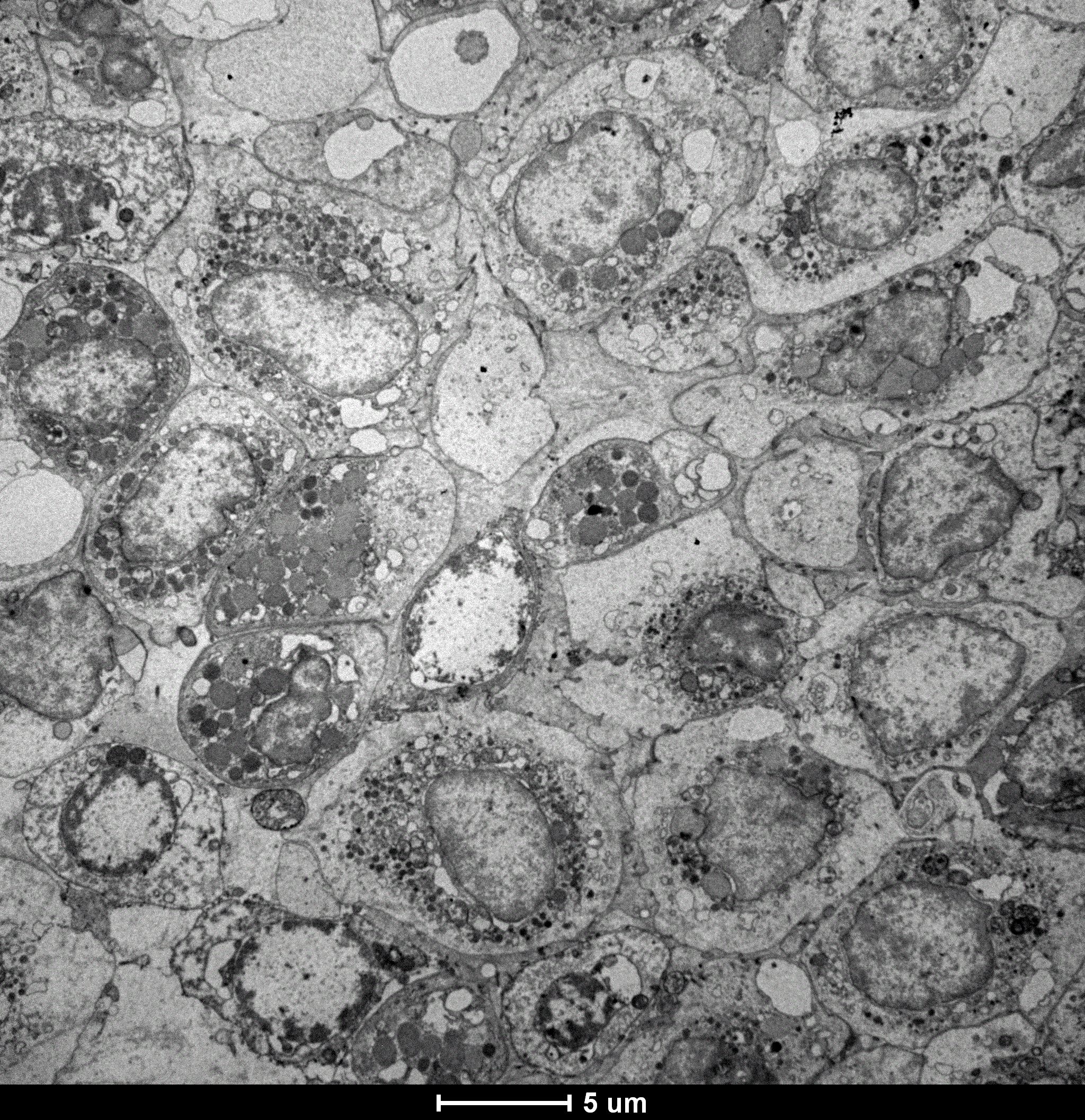

Supplement: Supplementary file 1 [file jox-16-00096-s001.zip › FigureS1-S3/Figure S1B.gif]

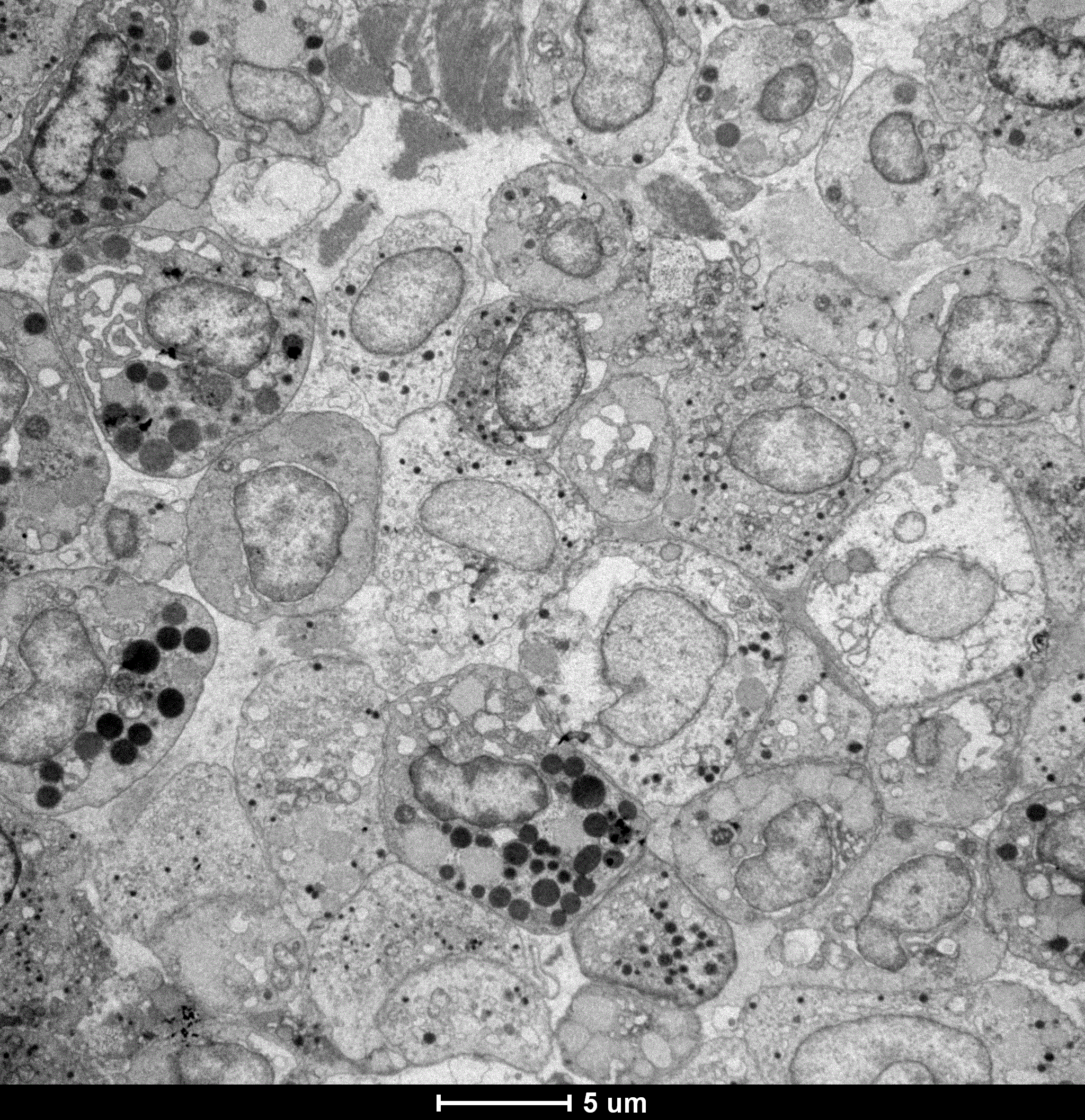

Supplement: Supplementary file 1 [file jox-16-00096-s001.zip › FigureS1-S3/Figure S1C.gif]

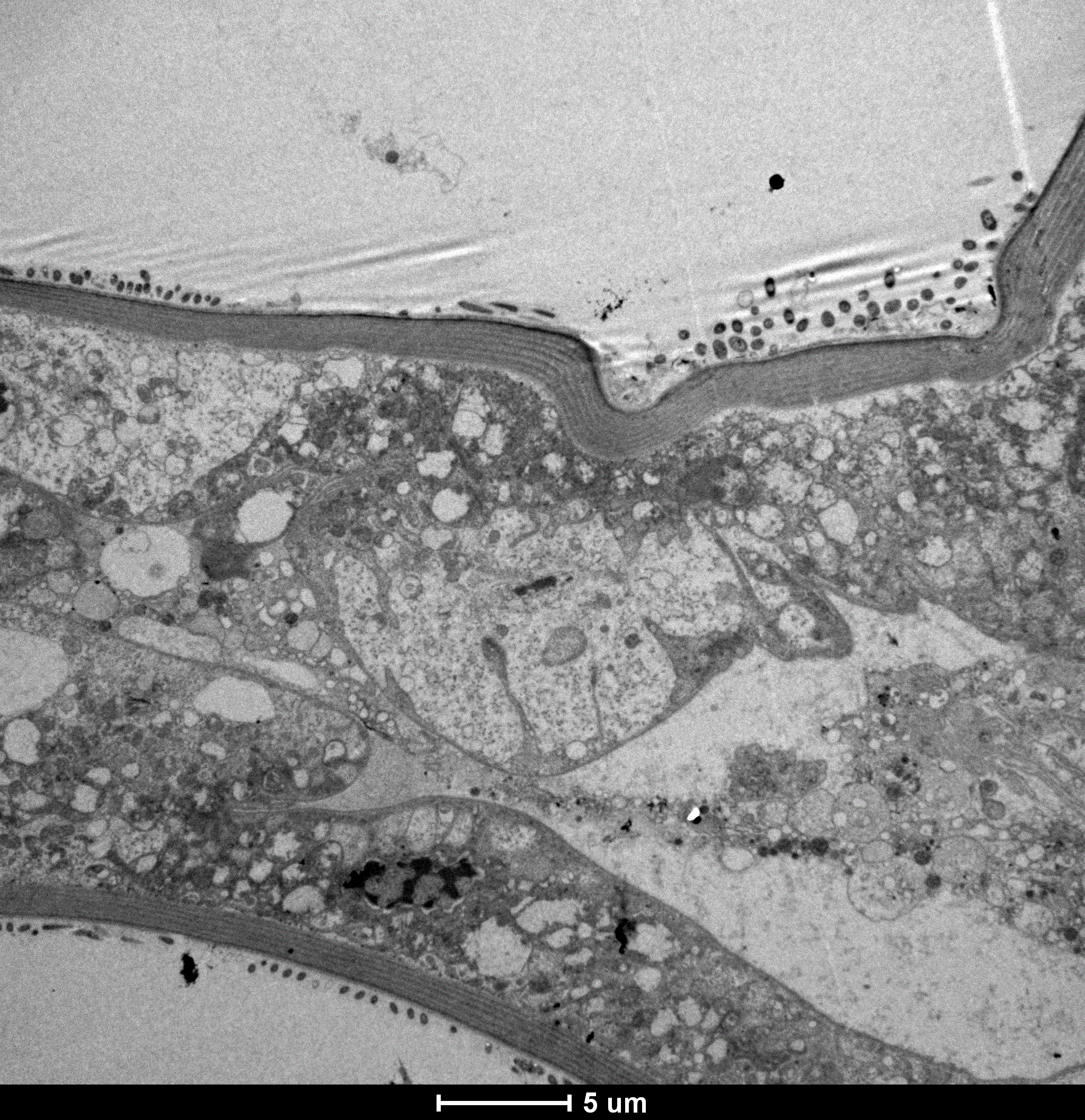

Supplement: Supplementary file 1 [file jox-16-00096-s001.zip › FigureS1-S3/Figure S2A.gif]

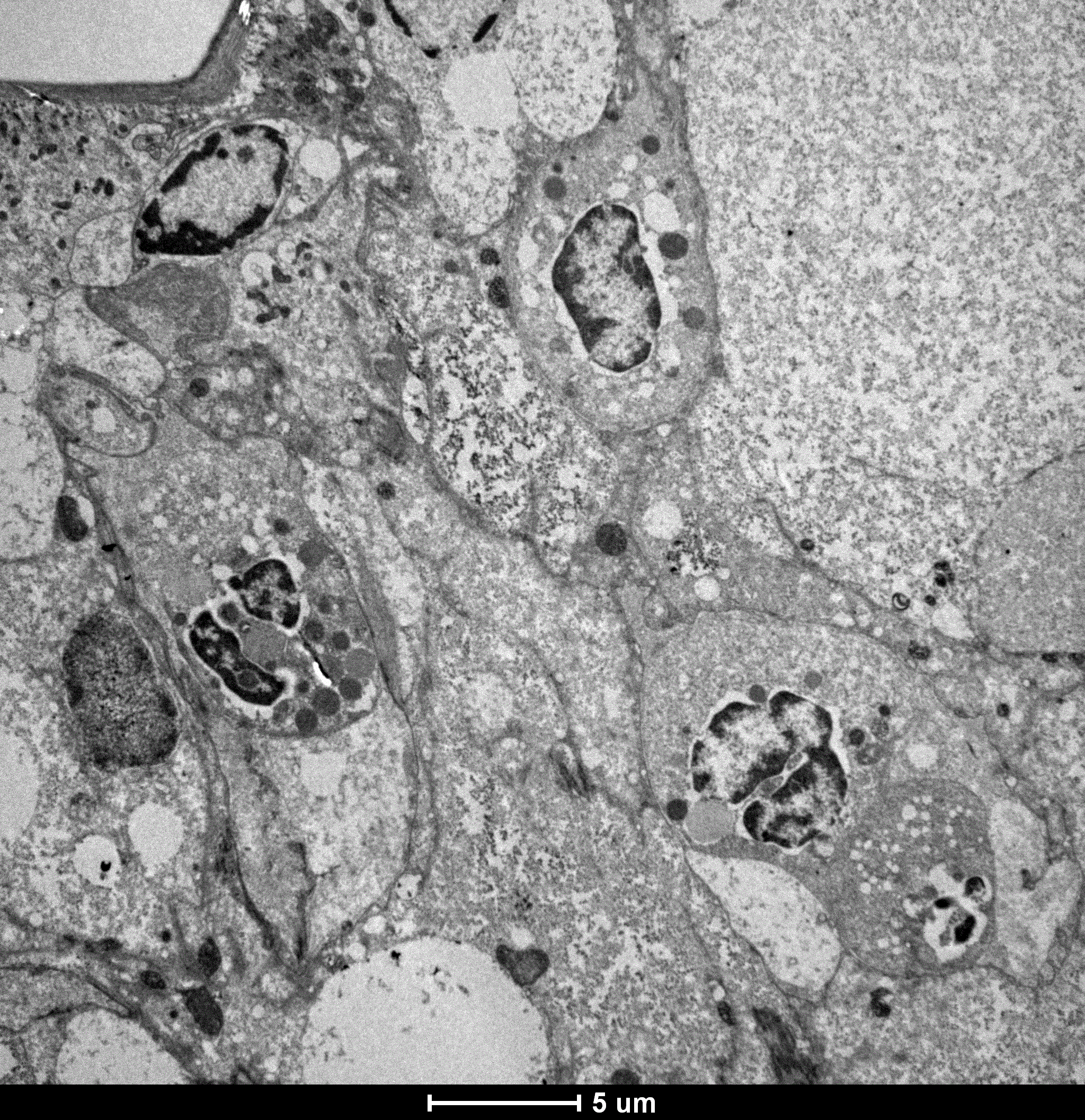

Supplement: Supplementary file 1 [file jox-16-00096-s001.zip › FigureS1-S3/Figure S2B.gif]

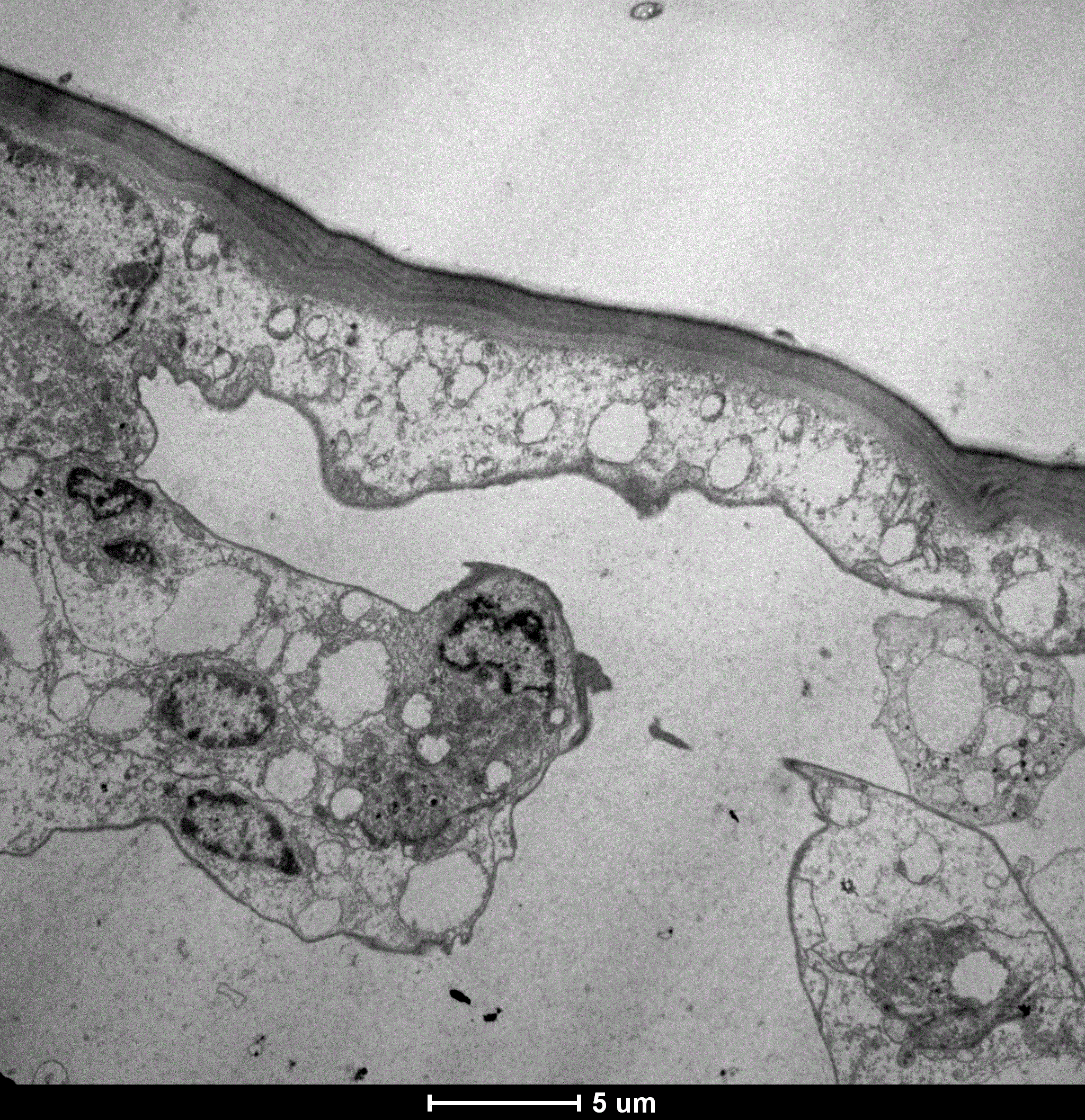

Supplement: Supplementary file 1 [file jox-16-00096-s001.zip › FigureS1-S3/Figure S2C.gif]

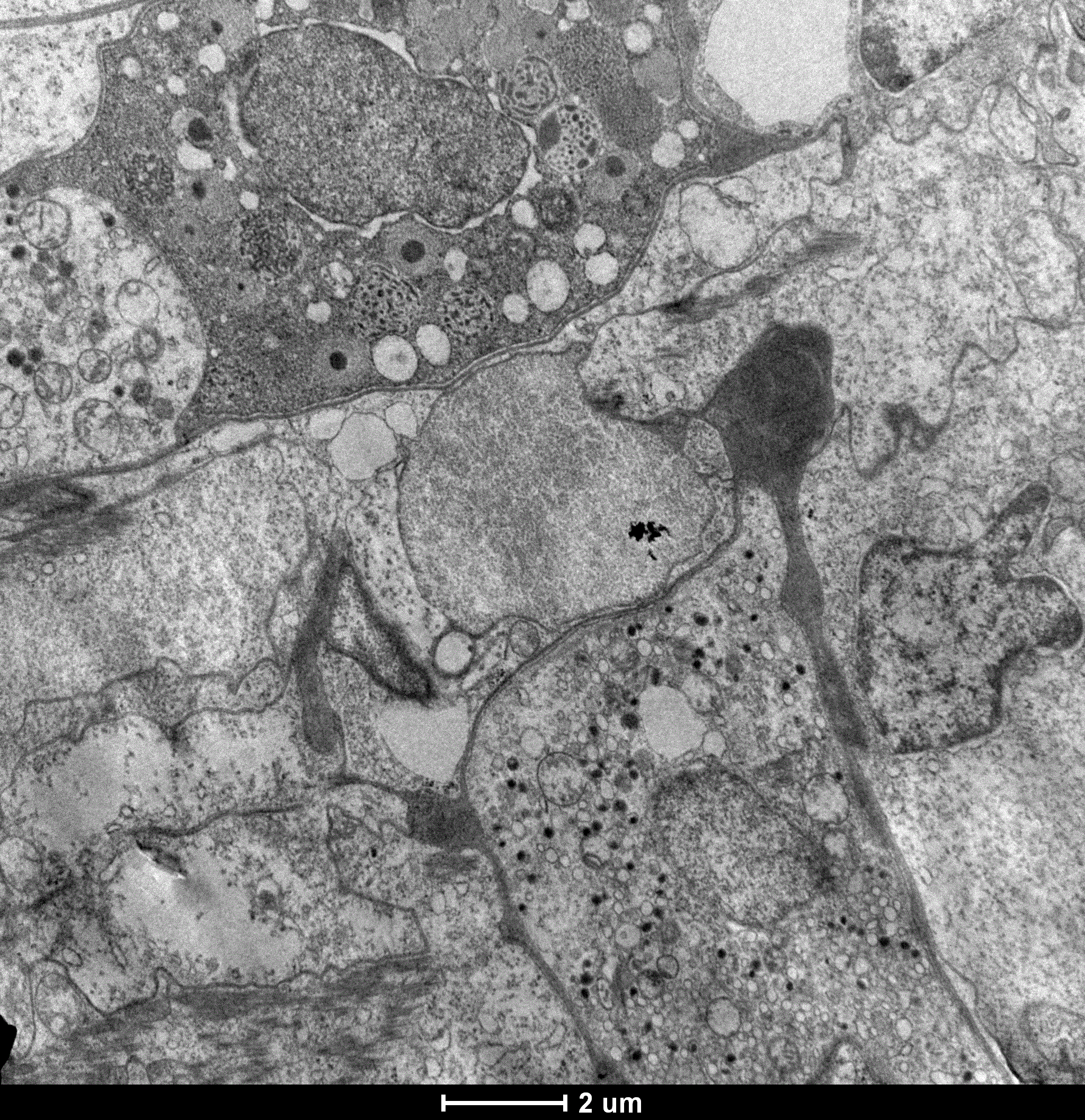

Supplement: Supplementary file 1 [file jox-16-00096-s001.zip › FigureS1-S3/Figure S2D.gif]

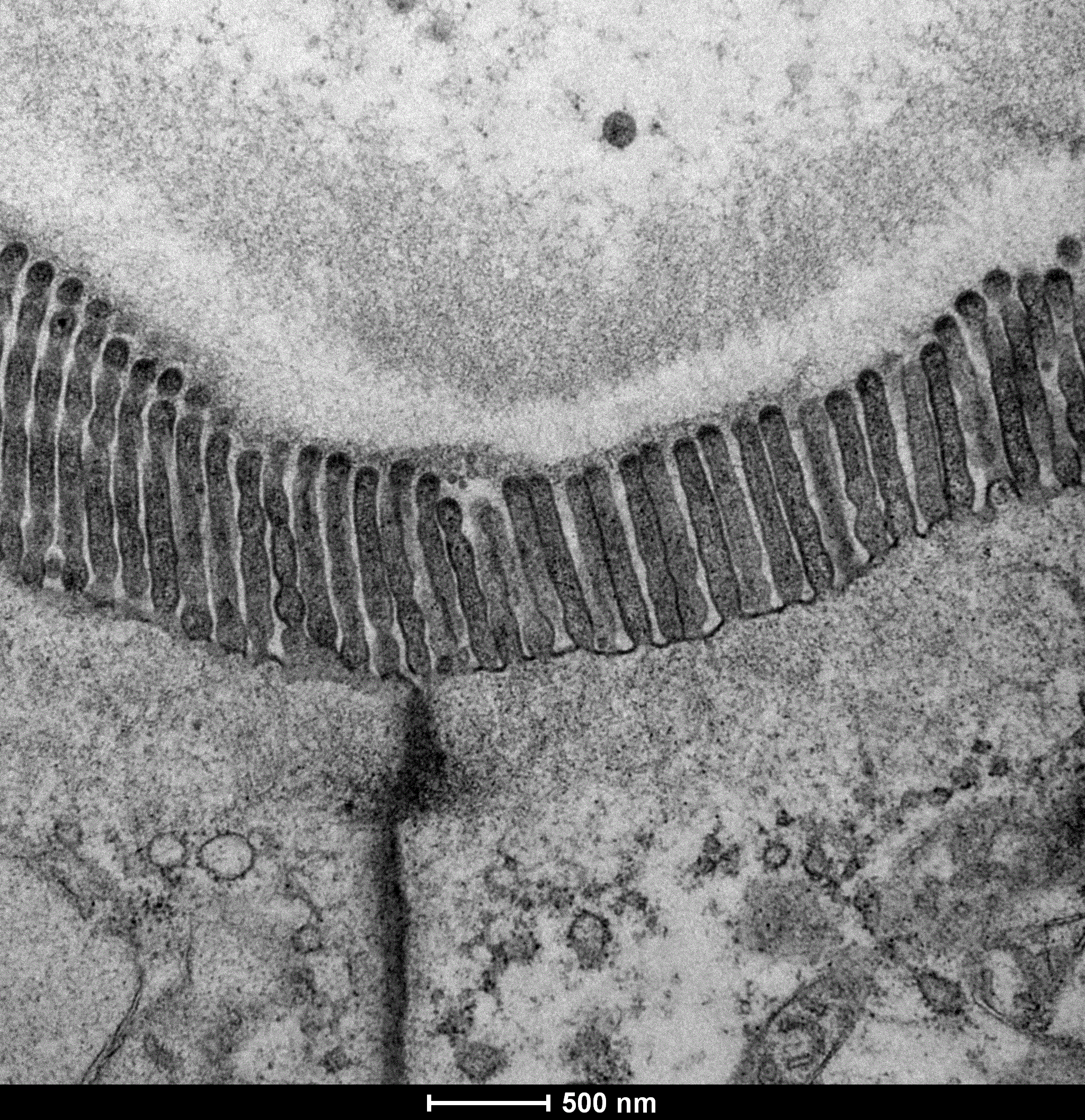

Supplement: Supplementary file 1 [file jox-16-00096-s001.zip › FigureS1-S3/Figure S3A.gif]

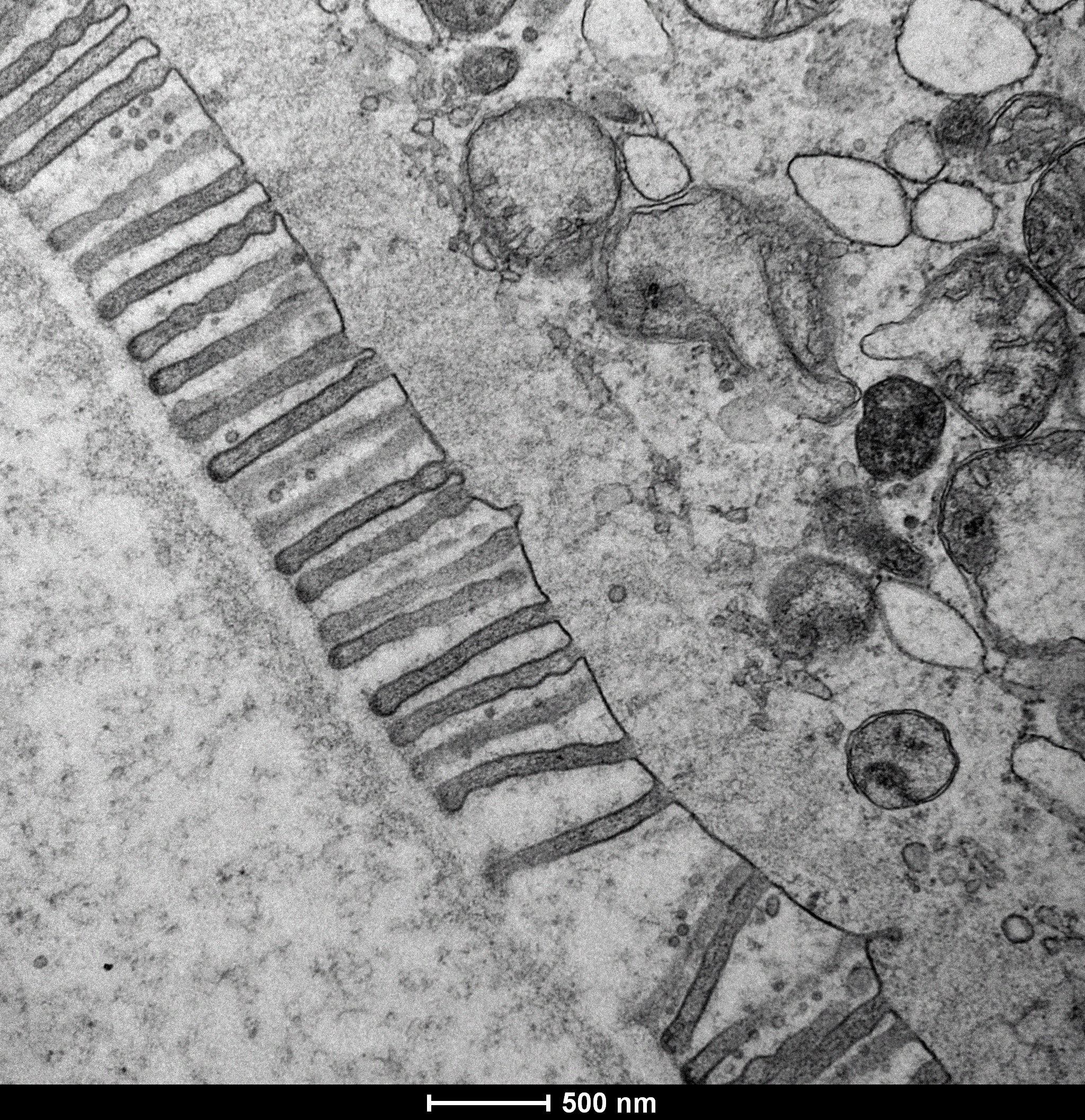

Supplement: Supplementary file 1 [file jox-16-00096-s001.zip › FigureS1-S3/Figure S3B.gif]

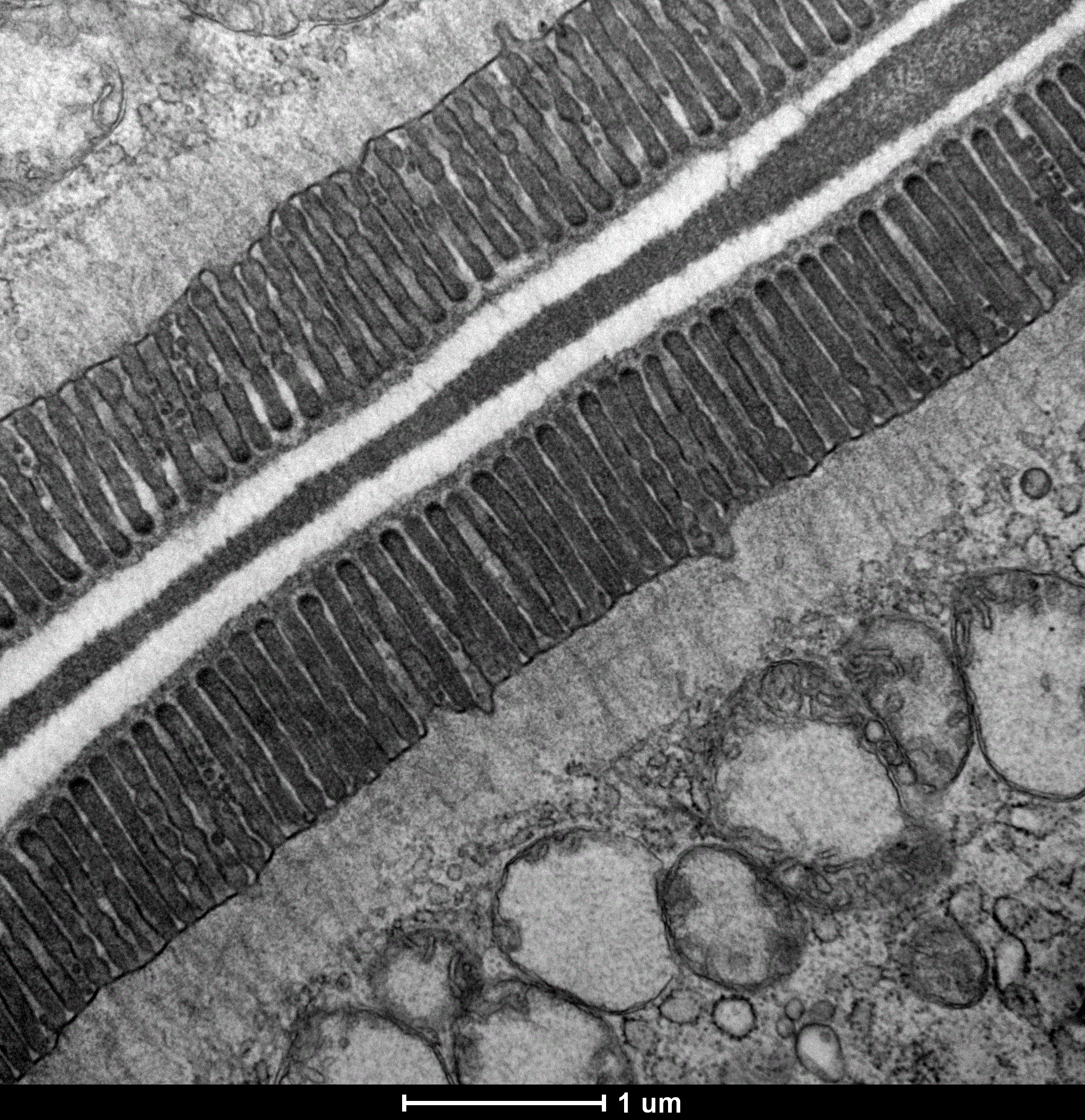

Supplement: Supplementary file 1 [file jox-16-00096-s001.zip › FigureS1-S3/Figure S3C.gif]

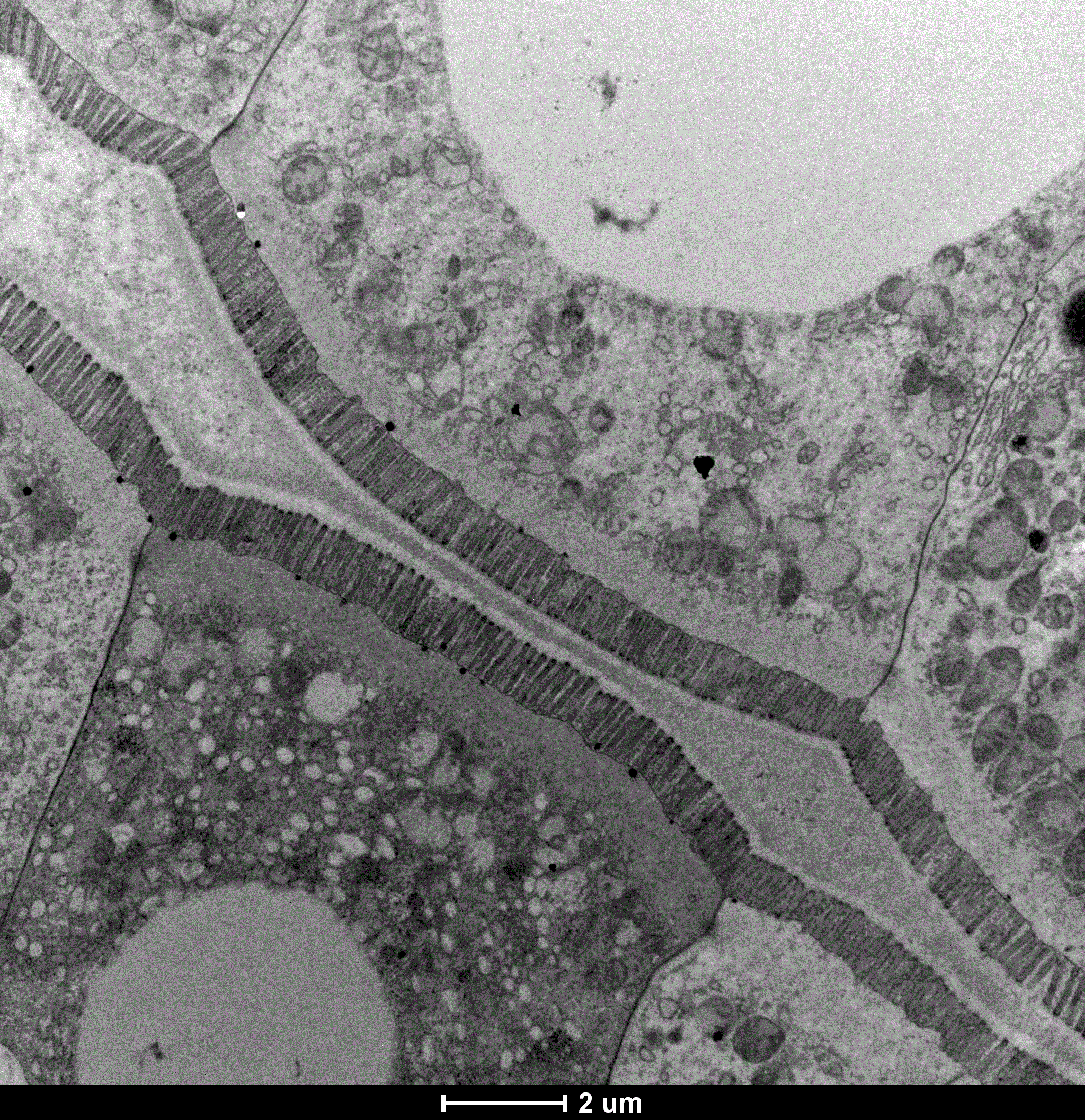

Supplement: Supplementary file 1 [file jox-16-00096-s001.zip › FigureS1-S3/Figure S3D.gif]
